# Recursive Partitioning Analysis of Lymph Node Ratio in Breast Cancer Patients

**DOI:** 10.1097/MD.0000000000000208

**Published:** 2015-01-09

**Authors:** Yao-Jen Chang, Kuo-Piao Chung, Li-Ju Chen, Yun-Jau Chang

**Affiliations:** From the Department of Surgery (Yao-Jen Chang), Taipei Branch, Buddhist Tzu Chi General Hospital; Graduate Institute of Health Policy and Management (K-PC, L-JC), College of Public Health, National Taiwan University; Department of Ophthalmology (L-JC), HepingFuyou Branch; Department of General Surgery (Yun-Jau Chang), Zhong-Xing Branch, Taipei City Hospital; and Department of General Surgery (Yun-Jau Chang), National Taiwan University Hospital, Taipei, Taiwan.

## Abstract

Lymph node ratio (LNR) is a powerful prognostic factor for breast cancer. We conducted a recursive partitioning analysis (RPA) of the LNR to identify the prognostic risk groups in breast cancer patients. Records of newly diagnosed breast cancer patients between 2002 and 2006 were searched in the Taiwan Cancer Database. The end of follow-up was December 31, 2009. We excluded patients with distant metastases, inflammatory breast cancer, survival <1 month, no mastectomy, or missing lymph node status. Primary outcome was 5-year overall survival (OS). For univariate significant predictors, RPA were used to determine the risk groups. Among the 11,349 eligible patients, we identified 4 prognostic factors (including LNR) for survival, resulting in 8 terminal nodes. The LNR cutoffs were 0.038, 0.259, and 0.738, which divided LNR into 4 categories: very low (LNR ≤ 0.038), low (0.038 < LNR ≤ 0.259), moderate (0.259 < LNR ≤ 0.738), and high (0.738 < LNR). Then, 4 risk groups were determined as follows: Class 1 (very low risk, 8,265 patients), Class 2 (low risk, 1,901 patients), Class 3 (moderate risk, 274 patients), and Class 4 (high risk, 900 patients). The 5-year OS for Class 1, 2, 3, and 4 were 93.2%, 83.1%, 72.3%, and 56.9%, respectively (*P*< 0.001). The hazard ratio of death was 2.70, 4.52, and 8.59 (95% confidence interval 2.32–3.13, 3.49–5.86, and 7.48–9.88, respectively) times for Class 2, 3, and 4 compared with Class 1 (*P* < 0.001). In conclusion, we identified the optimal cutoff LNR values based on RPA and determined the related risk groups, which successfully predict 5-year OS in breast cancer patients.

## INTRODUCTION

The lymph node ratio (LNR), defined as the number of positive lymph nodes (LNPs) divided by the number of total lymph nodes, has been advocated as one of the significant predictors in breast cancer patients.^1^ Many researchers have proposed that LNR may be an alternative staging system for prognosis because they observed that the LNR system predicted prognosis better than the traditional LNP system (the currently used pN1–3 classification is a categorization of the LNP system).^2–4^ Using the Cox proportional hazards model, they categorized LNR with several cutoff values to facilitate the incorporation of LNR into practical use in the near future. However, the cutoff values of LNR in the literature are either arbitrary or follow its predecessor's standards.^5–7^ Obviously, there is no consensus regarding which cutoff LNR values are the most reliable standard for predicting prognosis in breast cancer patients.^1^ In addition, it is possible that there are several factors rather than a single factor (such as LNR) in the real world that affect patient survival. Considering these factors, determining the prognostic LNR cutoffs have become meaningful and crucial. To this end, a risk group study may hopefully yield substantial information that is succinct and easily understood by researchers, providers, practitioners, and even policy makers to inform more appropriate choices.^8^

Risk groups can be determined using recursive partitioning analysis (RPA), which is intended to provide a way to divide patients into homogenous subsets based on the length of survival (or other dependent variables) and has the capacity to account for complex interactions among prognostic factors.^9,10^ As a multivariate technique, RPA provides a simple, straightforward, and intuitive method to classify subjects as well as to identify synergistic interaction among factors.^11,12^ It is considered a machine learning approach and usually requires a large data set to identify a classification model from a training sample and validate this model using a test sample. Although these calculations are complex, sophisticated computer systems offer a good solution for these tedious computations. Many health care studies have employed RPA to explore prognostic factors and risk groups, including those related to breast cancer in just a few cases.^12–14^

This study consisted of a 2-fold main objective. First, we sought to determine the most effective LNR cutoffs in patients from a population-based data set with noninflammatory breast cancer who underwent mastectomy. Second, we sought to identify the risk groups who were prognostic of overall survival (OS) based on RPA. We also compared these groups regarding 2 different types of survival (OS and cancer-specific survival [CSS]).

## MATERIALS AND METHODS

### Study Population

From the Taiwan Cancer Database (TCDB), which is a nationwide database, all patients (between 25 and 95 years old) with a primary diagnosis of breast cancer between January 2002 and December 2006 were candidates for the present study.^15^ The TCDB, released by the Department of Health, was also linked to another nationwide database, Death Registration (from the Ministry of the Interior), to retrieve individual causes of death. Exclusion criteria included stage 0 and IV disease, inflammatory breast cancer, pathology reports that did not include infiltrating ductal carcinoma, tumor size >5 cm, a lack of radical mastectomy or modified radical mastectomy, survival <1 month, and unknown lymph node status. We also excluded patients whose survival status could not be verified as of December 31, 2009. This study was approved by the Institutional Review Board at the Buddhist Xindian Tzu Chi General Hospital (No: 01-X07-028).

### Prognostic Variables

Before the RPA process in this study, a set of variables had been evaluated as prognostic factors: age (split at 45 and 65 years), tumor laterality (right breast vs left breast), tumor location (4 quadrants, central region, and overlapping quadrant), pathological tumor size (<2 cm, 2–5 cm), tumor cell grade (well differentiation, moderate differentiation, and poor differentiation), distance of surgical margin to tumor (positive or safety margin <0.2 cm, 0.2–1 cm, >1 cm), number of lymph nodes retrieved and examined (LNT), LNPs, LNR, TNM stages (stage I, stage II, and stage III, based on the classification of the 6th edition of the *American Joint Commission's Cancer Staging Manual*), status of hormone receptor (negative vs positive), chemotherapy (yes vs no), radiotherapy (yes vs no), and hormone therapy (yes vs no).

### Study Outcomes

The primary end point was 5-year OS. The OS rate referred to the percentage of patients who were still alive for a certain period of time after breast cancer surgery. The secondary end point was 5-year CSS. The CSS rate referred to the percentage of patients who had not died from breast cancer or metastasis for a certain period of time after breast cancer surgery.^15^ Survival was calculated from the day of surgery (modified radical mastectomy).

### Statistical Analysis

Univariate survival analyses were constructed for OS to identify significant factors. Then, RPA was used to define the breast cancer risk groups. Several partitioning techniques were available to conduct RPA, including Chi-Square Automatic Interaction Detector (CHAID), QUEST (Quick-Unbiased-Efficient Statistical Tree), and Classification and Regression Trees (CRT).^16^ These techniques belong to a nonparametric methodology that creates a decision tree (or survival tree) with respect to prognostic factors and their interactions that are most important in determining the outcome. We chose the CRT technique in the process of risk group construction because it was very easy to understand. During the process of partitioning, we randomly assigned 50% of the cohort as the training sample and the remaining 50% of the cohort as the test sample to reduce overfitting and upward-biased estimates of the coefficients. The criteria for splitting included the following: child nodes derived from a parent node should be as homogeneous as possible with the dependent variables (eg, OS); corresponding cut off points should result in the minimal *P* value, provided the minimal *P* value was ≤0.0001; the number of patients within the child node should be at least 50.^8,16^ Terminal nodes, if any, would be identified after completion of the split process and could be assigned to a class when the significance level of comparison between 2 terminal nodes was >0.05.^8^ The Kaplan–Meier method was used to calculate cumulative survival regarding these RPA classes. Survival differences among RPA classes were tested using the log-rank test, and the hazard ratios (HRs) and 95% confidence intervals (CIs) were reported. Statistical software PASW Statistics 18 (SPSS Inc., Chicago, IL, USA) was used for all of the analyses reported in this study, and *P* values of <0.05 were considered statistically significant.

## RESULTS

Among 27,754 newly diagnosed breast cancer candidates, there were 11,349 patients who had undergone mastectomy for breast cancer and met the inclusion criteria. Receiving treatment at 32 hospitals or cancer centers, these patients were enrolled in this survival tree analysis. According to tumor stage of these enrollees, 26.6%, 52.7%, and 20.7% of patients presented with stage I, stage II, and stage III disease, respectively (Table 1 ). Fifty-two percent of breast cancer arose in the left breast. Pathology reports showed that 1.4% of patients had a safety margin <0.2 cm. Ninety percent of the patients had at least 10 lymph nodes examined and 46.8% of patients had metastatic lesions in the examined lymph nodes. There were 63.8% and 56.6% of tumors presenting with positive estrogen receptor (ER-positive) and positive progesterone receptor (PR-positive). Regarding adjuvant treatment, 73%, 20.3%, and 62.3% of patients received chemotherapy, radiotherapy, and hormone therapy, respectively. The duration of follow-up was 1 to 96 months, with a mean of 57.5 (±18.1) months. The *P* value of log-rank test for all factors was <0.001 (except laterality, location, and chemotherapy, *P* = 0.178, *P* = 0.141, and *P* = 0.210, respectively) in univariate survival analysis. For chemotherapy, however, we noted that the associations were significant when stratified by pathologic stage (for stage II, HR = 0.68, 95% CI 0.55–0.83, *P* < 0.001; for stage III, HR = 0.53, 95% CI 0.43–0.67, *P* < 0.001). All significant predictors (including chemotherapy) were included in the partitioning process. Concerning multicollinearity, we excluded LNT, LNP, and tumor stage (LNT, LNP were highly correlated with LNR, coefficient of correlation >0.80; tumor stage was strongly correlated with LNR, coefficient of correlation = 0.66) because they were correlated with LNR.

**TABLE 1:**
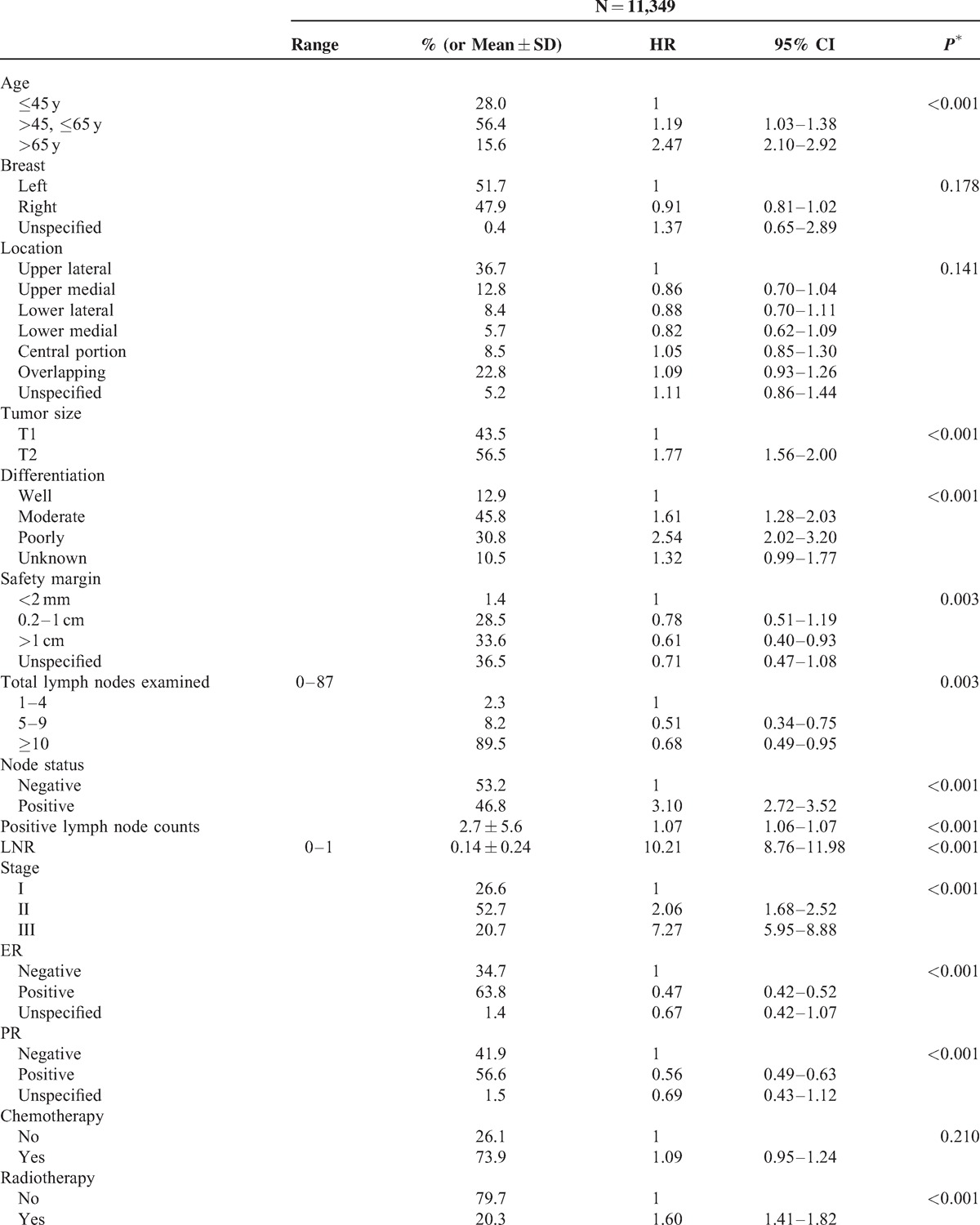
Univariate Analyses of Clinical, Pathological and Treatment Factors in Breast Cancer

**TABLE 1 (Continued):**
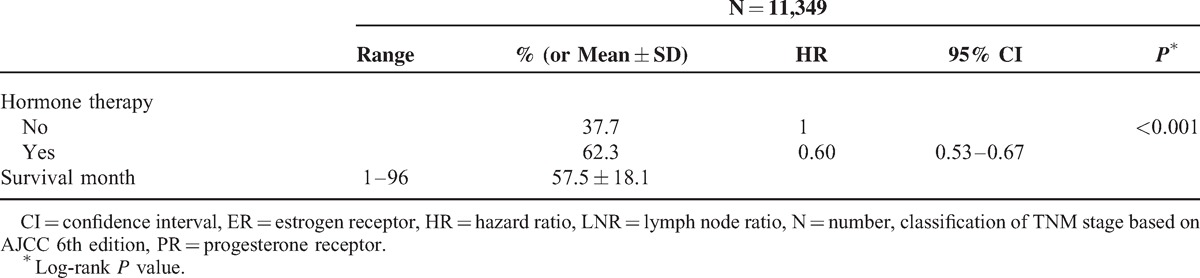
Univariate Analyses of Clinical, Pathological and Treatment Factors in Breast Cancer

**FIGURE 1:**
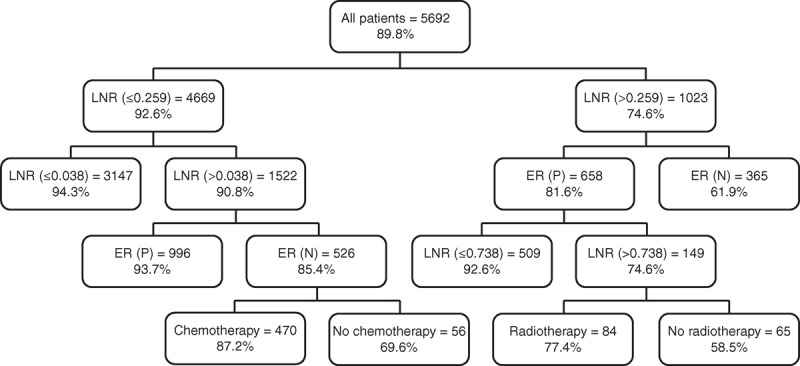
Results of the classification and regression tree (training sample) were obtained from 5,692 breast cancer patients who underwent modified radical mastectomy. The number in the upper half of the box indicates the number of patients; the percentage in the upper half of the box indicates OS percentage. OS = overall survival, ER (N) = estrogen receptor (negative), ER (P) = estrogen receptor (positive), LNR = lymph node ratio.

The recursive partitioning process was started with a training sample of 5,692 patients in which 581 patients (10.2%) had died during the study period. Because LNR was the most important factor, the partitioning process initially yielded 4,669 patients (6.9% dead) with LNR ≤ 0.259 and 1,023 patients (25.4% dead) with LNR > 0.259 (*P* < 0.001). The same process was continued following these splitting criteria (Figure 1). In the left panel, LNR appeared to be the strongest factor (*P* < 0.001), which yielded a subgroup of 3,147 patients with LNR ≤ 0.038 (5.7% dead) and a subgroup of 1,522 patients with LNR > 0.038 (9.2% dead) (*P* < 0.001). No further split was possible in the node of LNR ≤ 0.038 due to minimal criteria. The node with 0.038 < LNR ≤ 0.259 was split into a subgroup of 996 patients who were ER-positive (6.3% dead) and a subgroup of 526 patients who were PR-negative (14.6% dead) (*P* < 0.001). No further split was possible in the ER-positive node due to minimal criteria. However, the node with 0.038 < LNR ≤ 0.259 and PR-negative was split into a subgroup of 470 patients who had received chemotherapy (12.8% dead) and a subgroup of 56 patients who had not received chemotherapy (30.4% dead), both of which were terminal nodes.

**FIGURE 2:**
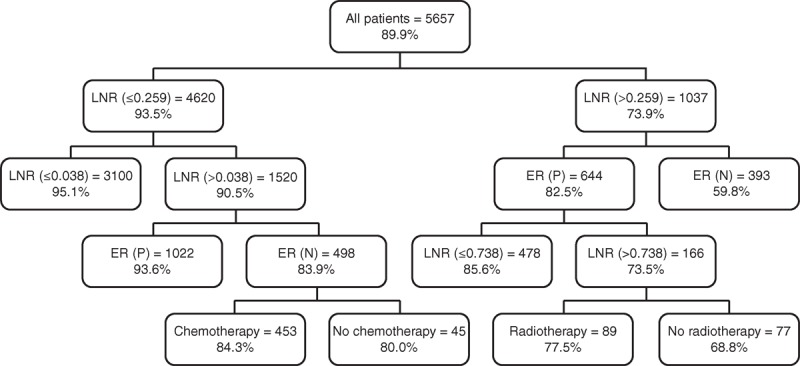
Results of the classification and regression tree (test sample) were obtained from 5,657 breast cancer patients who underwent modified radical mastectomy. The number in the upper half of the box indicates the number of patients; the percentage in the upper half of the box indicates OS percentage. OS = overall survival, ER (N) = estrogen receptor (negative), ER (P) = estrogen receptor (positive), LNR = lymph node ratio.

In the right panel (Figure 1), ER status was the strongest factor and yielded a subgroup of 658 ER-positive patients (18.4% dead) and a subgroup of 365 ER-negative patients (30.1% alive) (*P* < 0.001). The node of ER-negative patients and LNR > 0.259 was a terminal node because no further split was possible. The node of ER-positive patients and LNR > 0.259 was further split into a subgroup of 509 patients who were ER-positive with a LNR between 0.259 and 0.738 (7.4% dead) and a subgroup of 149 patients who were ER-positive with a LNR > 0.738 (25.4% dead) (*P* < 0.001). No further split was possible in the node of ER-positive and 0.259 < LNR ≤ 0.738 due to minimal criteria. The node of ER-positive patients and LNR > 0.738 was further split into a subgroup of 84 patients who received radiotherapy (22.6% dead) and a subgroup of 65 patients who did not receive radiotherapy (41.5% dead) (*P* < 0.001), both of which were terminal nodes. The results of the training sample were validated with a test sample of 5,657 breast cancer patients, which were independent of the model building training sample (Figure 2). Both results were closely correlated.

Following this process, 4 prognostic factors were identified (LNR, ER status, chemotherapy, and radiotherapy) for OS, evolving into 8 terminal nodes. Then, we divided these terminal nodes into 4 risk groups according to mean survival time (Table 2). Based on LNR values of 0.038, 0.259, and 0.738, 4 levels of LNR (very low [≤0.038], low [between 0.038 and 0.259], moderate [between 0.259 and 0.738], high [>0.738]) were defined for description. Class 1 (very low risk) consisted of 8,265 patients who had very low LNR or who had low LNR and were ER-positive (461 deaths during the study period). Class 2 (low risk) consisted of 1,910 patients who had moderate LNR and were ER-positive or who had low LNR, were ER-negative, and were treated with chemotherapy (275 deaths during the study period). Class 3 (moderate risk) consisted of 274 patients who had low LNR, were ER-negative, and were not treated with chemotherapy or who had high LNR, were ER-positive, and underwent radiotherapy (65 deaths during the study period). Class 4 (high risk) consisted of 900 patients who had high LNR, were ER-positive, and did not undergo radiotherapy or who had moderate to high LNR and were ER-negative (348 deaths during the study period).

**TABLE 2:**
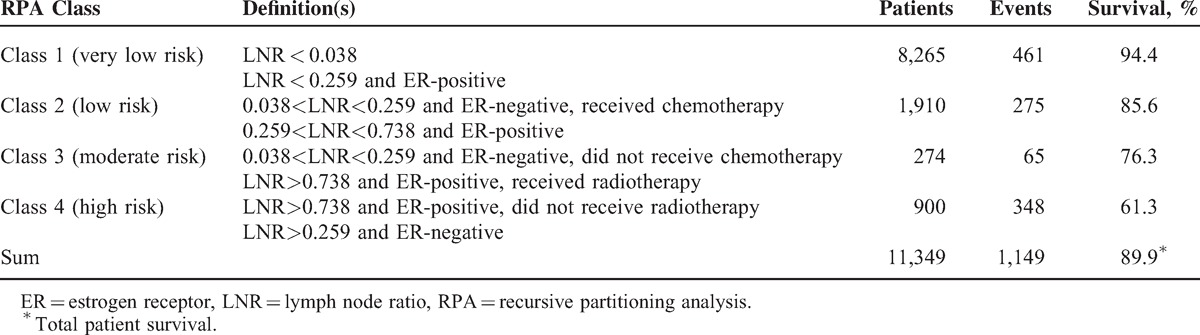
Assignment of Risk Groups According to RPA

**FIGURE 3:**
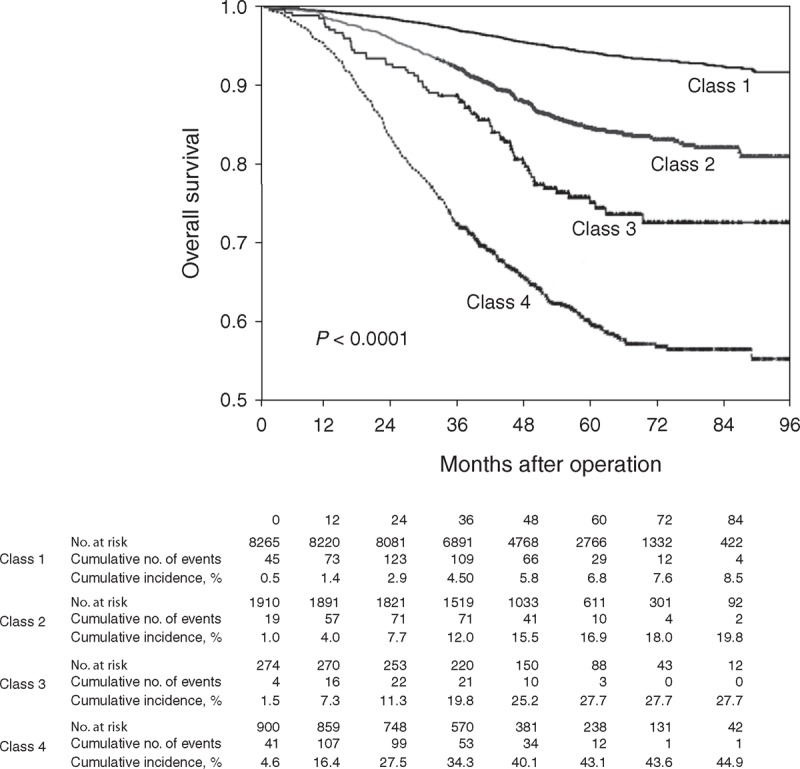
Kaplan–Meier OS estimates in breast cancer patients according to RPA classes. OS = overall survival, RPA = recursive partitioning analysis.

According to this risk classification, Kaplan–Meier plot and log-rank test were applied to demonstrate differences in OS among each class (*P* < 0.0001, Figure 3). The results showed that these risk groups had good discriminating capability to predict patient outcomes. Classes 2, 3, and 4 had significantly poorer 5-year survival probability (HR 2.70, 4.52, and 8.59; 95% CI 2.32–3.13, 3.49–5.86, and 7.48–9.88, respectively, see Table 3) compared with Class 1. In addition, we tested these RPA classes to predict CSS, which in turn yielded similar results and higher HR values.

**TABLE 3:**
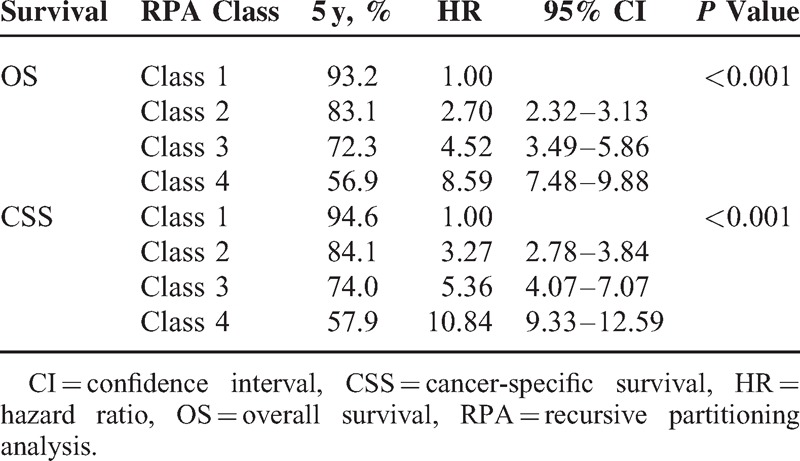
RPA Groups and Survival

## DISCUSSION

In this decision tree model (RPA) of breast cancer patients treated with mastectomy and lymph node dissection, we identified 4 risk classes that have prognostic significance in OS and CSS. Each of the RPA classes exhibited approximately 10% discrepancy in the 5-year OS (and CSS) compared with neighboring RPA classes. Although risk group definition based on RPA has been widely accepted and adopted to predict the outcomes of several benign and malignant diseases^10,14,17–19^, it is still not commonly used in the field of breast cancer care. A possible reason is that most surgeons, especially breast cancer surgeons, are not acquainted with this simple method although it requires statistical software to perform the cumbersome calculations. Introducing this decision tree model may allow decisions (such as those based on LNR cutoffs) that were previously difficult to be more easily made given that this model solves the interaction problem.

The other finding of this study was that RPA yielded 3 LNR cutoffs (0.038, 0.259, and 0.738) with prognostic significance. Therefore, we stratified LNR into 4 levels: very low, low, moderate, and high level. In a study of 1,829 breast cancer patients, Vinh-Hung et al^4^ identified optimal LNR cutoffs of 0.2 and 0.65, which were derived from difference computations of model likelihood and Akaike Information Criteria based on an LNR interval of 0.05 (ranging from 0.05 to 0.95) and bootstrapping. To date, there are few large scale studies (>3,000 breast cancer patients, follow-up >5 years) that have investigated the prognostic significance of LNR in breast cancer patients using stratified groups according to cutoff LNR values. Four population-based studies including at least 17,000 breast cancer patients followed this cutoff standard and demonstrated the prognostic utilities of such categorization.^5,20–22^ Recently, a large study of 7,741 breast cancer patients was published by Kim et al^23^ who recommended that the best LNR cutoff values were 0.18 and 0.64 using a hazards model by examining information loss with an increment of 0.001 internal (ranging from 0.001 to 0.85). Studies with relatively smaller sample sizes stratified patients based on either the standard of 0.2 and 0.65^24,25^ or the quartile rule,^26^ empirical experience^27^ or based on groups that were homogenously distributed.^28^

In contrast to this finding of 2 cutoff LNR values in breast cancer patients, our study demonstrated an additional cutoff at 0.038. The most likely reason is that the original computation of the optimal LNR interval of 0.05 may overlook the observation of important cutoffs when smaller LNR intervals are needed. It also highlights the advantage of machine learning techniques, such as RPA, which do not require predefined assumptions and are especially suitable with continuous variables (eg, LNR). Previous researchers did not use 3 cutoff values for LNR classification except one who applied mathematics quartiles (LNR 0.25, 0.50, and 0.75) to 669 breast cancer patients and demonstrated their predictive capabilities.^26^ It raises an important issue of the most important objective that the cutoff value predicts. Evidence shows that as a continuous variable, LNR carries a statistically prognostic significance, and any type of LNR categorization will certainly achieve prognostic goals, similar to traditional LNP classification. We present here an attempt to illustrate how the categorization of LNR can be applied to daily practice (such as adjuvant chemotherapy or radiotherapy) because such a classification related to therapy would deliver more benefits to cancer care. Determining the complementary N stage, after all, should not only be restricted to the definition of disease severity but also should aim to provide professionals with information for planning appropriate treatment.

The visually succinct expression of RPA allows the easy and quick understanding of a complicated prognostic model.^14^ Displaying the information in a decision tree model allows readers to understand the importance of specific factors and relationships of factor interaction at a first glance. For example, our results showed that LNR was the most important in this model, followed by ER status. Chemotherapy is indicated for breast cancer patients with low LNR who are ER-negative. Radiotherapy is indicated for breast cancer patients with moderate LNR who are ER-positive. In the literature, there is no related report on LNR interval ranges in which chemotherapy is recommended. Nonetheless, a study conducted by Kim et al^23^ showed that postmastectomy radiotherapy provided no survival benefit for breast cancer patients with pN1 disease when the LNR was <0.18. They suggested postmastectomy radiotherapy for pN1 patients with LNR values between 0.18 and 0.64. Similarly, in patients with pN1 disease, Huang et al^29^ suggested postmastectomy radiotherapy for patients with LNR values >0.25 to reduce locoregional recurrence and to improve disease-free survival. Both studies showed a little difference from the conclusion of a meta-analysis conducted by Li et al,^30^ which indicated that postmastectomy radiotherapy significantly decreased the risk of local recurrence in 3,432 breast cancer patients with pN1 disease.

Although multivariate analyses of the Cox proportional hazards model can identify a certain prognostic or risk factor and can compute the hazard ratio for an entire population, it does not automatically investigate the interaction terms. Instead, RPA allows different prognostic factors for different branches of the model. RPA is a statistic methodology that is suitable when there is interaction between risk factors and when there are continuous variables requiring optimal cutoff values.^8^ In addition to binary split decisions of CRT technique, the multiple split decision algorithms are available. The optimal LNR cutoff values can also be determined with the CHAID technique, which constructs a decision tree by repeatedly splitting subsets into 2 or more child nodes, beginning with the entire group.^31^ Indeed, several alternative methods other than RPA are available for determining the optimal LNR cutoff values, such as receiver operating characteristic curve, minimization of *P* values, and running log rank test.^32–34^ Authors can use these methods based on the appropriate rationale according to the purpose of their study.

The current study made 2 contributions toward predicting outcomes in breast cancer patients after mastectomy. First, we applied classification and regression models to predict survival in breast cancer and defined the risk groups related to long-term survival. To the best of our knowledge, this is the first report regarding tree-structured survival analysis in breast cancer patients using population-based data. Second, we identified LNR cutoff values that were prognostic for survival in relation to hormone receptors and adjuvant therapy, which has never been reported in the literature. This allows us to extend the oncology research into a method that addresses the issue of interaction very effectively.

We acknowledge that our study suffers from several shortcomings, including a short follow-up time. Several large-scale studies exceeded 10 years of follow-up. In addition, we did not include any HER-2/neu status in the RPA process due to unavailability of the data. As a population-based database, TCDB does not regularly collect information on HER-2/neu status in breast cancer patients. Fortunately, we could add the hormone receptor (ER and PR) status and adjuvant therapy information (chemotherapy, radiotherapy, and hormone therapy) into our statistics models. Additionally, the inherent 3 month interval of the TCDB update prevented us from pursuing correct recurrence status in the breast cancer patients, which might have been an important end point when we tried to determine the optimal LNR cutoffs. Finally, this population-based data set covered only approximately 60% of the breast cancer patients who are newly diagnosed in Taiwan annually, and therefore, the results may not be generalizable to the entire nation. However, we think that this data set can represent daily practice that is encountered every day. In particular, the health care insurance system in Taiwan is a single-payer system, and medical expenses are under regular audit.

## CONCLUSIONS

In conclusion, the current study defined 4 risk groups in breast cancer patients after mastectomy by employing RPA. These 4 risk groups correspond to 5-year OS and CSS rates that are statistically significant. Four prognostic factors were determined, among which, LNR was the most important, followed by the ER status. We also identified 3 LNR cutoff values, with which the risk groups of breast cancer patients were determined. RPA acts as an alternative method of categorizing LNR, particularly when we consider incorporating the interaction term to provide a helpful guide to decision making in treatment.
